# Quinolone-Induced Painful Peripheral Neuropathy: A Case Report and Literature Review

**DOI:** 10.1177/2324709617752736

**Published:** 2018-02-26

**Authors:** Leonel J. F. Estofan, Stanislav Naydin, Gediminas Gliebus

**Affiliations:** 1 Drexel Neurosciences Institute, Philadelphia, PA, USA

**Keywords:** diabetes mellitus, levofloxacin, IVIG, peripheral neuropathy

## Abstract

We present a case report of a 20-year-old male with diabetes mellitus type 1, who developed severe painful peripheral neuropathy while on the second of a 10-day course with levofloxacin for the treatment of epididymitis. The intensity of the pain rapidly reached scores of 10/10 in a numeric scale 0/10, and the patient was transferred to an inpatient pain unit where he was treated aggressively with minimal improvement. A skin biopsy revealed small fiber neuropathy. Then the patient was treated with intravenous immunoglobulin, which improved the pain. Now the patient is on outpatient intravenous immunoglobulin infusions bimonthly and making a slow recovery.

## Introduction

Levofloxacin is an antibiotic utilized to treat several types of bacterial infections including those of the genitourinary tract. Also, it has been associated with tendinitis and spontaneous tendon rupture. Peripheral neuropathy has been included in the Black Box warning on fluoroquinolones by the US Food and Drug Administration (FDA). Peripheral neuropathy is a condition that can become chronic, is debilitating, and on occasions severe. The underlying mechanisms responsible for the neuropathy, risk factors, and course are still unclear. We report a case of levofloxacin-induced peripheral neuropathy in a patient with diabetes mellitus type 1.

## Case Presentation

**Figure 1.**
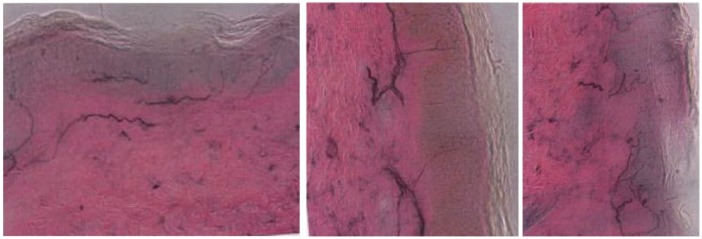
*Left distal leg*: The image demonstrates an area with low normal epidermal nerve fiber density. *Left foot*: The image demonstrates an area with normal epidermal nerve fiber density. *Left proximal thigh*: The image demonstrates an area with normal epidermal nerve fiber density.

We present the case of a 20-year-old Caucasian male with a history of type 1 diabetes mellitus, with an HbA1C of 9, who was seen by his endocrinologist 17 days before a hospital admission with a chief complain of scrotal pain and swelling. The endocrinologist referred the patient to his family doctor. Urinalysis suggested infection, and based on the clinical presentation, he was treated for suspected epididymitis with levofloxacin 500 mg daily for 10 days due to the patient’s allergy to penicillin including cephalosporin. Two days after starting the levofloxacin the patient began complaining of severe lower abdominal and back pain that radiated to the scrotum. The family attributed the back pain to physical exhaustion caused by his physically demanding job at a granite yard. The following day, the pain increased in intensity prompting the patient to go the emergency department (ED). In the ED, a scrotal ultrasound was performed to rule out testicular torsion, which was unremarkable. His pain was treated with Percocet, and he was discharged home on gabapentin 300 mg PO (per os) twice a day and Percocet. Approximately 2 days later the patient woke up in excruciating pain, screaming and complaining of feet and body pains that he described as burning. During the 10-day course of levofloxacin, the patient had a total of 3 visits to the ED at a community hospital for worsening generalized body pains that was more intense in the feet, which improved mildly with Percocet. He described his symptoms as muscle tightness, tendon pain above and below elbow, and knee joints. He also described coldness in the feet, weakness in the lower extremities, mid-back pain, tingling sensation in his extremities, and particularly a burning sensation from the knees down to his feet. Approximately 2 days after completing his 10-day levofloxacin course, the patient’s generalized body pains worsened, and he was noticed to have a wobbling gait, diffuse diaphoresis, and worsening temperature dysregulation. His family retook him to the ED, but this time he was admitted to pain management. At the fifth day, due to worsening of the symptoms, he was transferred to a teaching hospital for more aggressive management and diagnostic workup. At admission, his weight was 51.8 kg, the HbA1C was 9.6, and his glucose level was 158 mg/dL. His endocrinologist managed his insulin. The patient was on the following medications: insulin detemir 12 unit at bedtime; insulin lispro 4 units before breakfast, 8 units before lunch, and 11 units before dinner; gabapentin 300 mg oral bid (twice a day); and Percocet PRN (when needed) that was continued. Vitals were as follows: blood pressure 145/95 mm Hg, temperature of 98.1°F, heart rate of 118 beats per minute, and respiratory rate 18 breaths per minute. The neurological examination was significant for diffuse weakness in the lower extremities more distal than proximal; deep tendon reflex was 2/4 and symmetric at the biceps, triceps, knees, but decreased in the ankles; plantar responses were flexor; light touch and pinprick revealed hyperalgesia. He also had diffuse paresthesia that were more prominent in the lower extremities than upper extremities; proprioception sense and vibration were within normal limits; coordination was unremarkable; there were no abnormal or extraneous movements; and gait was unable to be assessed at that time due to pain in the sole of the feet. During the hospitalization, the patient’s pain became localized to the lower legs, below the knees, with an excruciating burning sensation accompanied by allodynia in the sole of the feet. To avoid contact with the sheets, he slept sitting up with his legs dangling from the bed (see Figure 1).

During the admission, a large battery of tests was conducted. A skin punch biopsy showed small fiber neuropathy. All other tests were negative and included the following: complete blood count, electrolytes, liver enzymes, sedimentation rate, C-reactive protein, vitamin B_12_, folate, heavy metals, porphyria, gangliosides, syphilis markers, Lyme antibodies, thyroid-stimulating hormone levels, paraneoplastic panel, immune-fixation, complement, hepatitis panel, electromyography/nerve conduction velocity, and magnetic resonance imaging of the brain and cervical spine.

The patient underwent trials with lidocaine infusion, Dilaudid PCA (patient controlled analgesia), methadone, opioids oral, ketamine infusion, and Tylenol intravenous, with a temporary improvement of the pain. At that point, we started an infusion with intravenous immunoglobulin (IVIG) 2 g/kg in divided doses over 3 days, and the pain decreased to 1/10 allowing the patient walk for the first time in over 6 weeks. The patient continued outpatient IVIG infusions, with pain scores that ranged 4/10 over the following 6 months.

## Discussion

We report a case of a 20-year-old male with diabetes mellitus who developed chronic small fiber neuropathy following treatment with quinolones. Based on this case study, we recommend avoiding prescribing fluoroquinolones in patients with type 1 diabetes mellitus that may be at risk to develop peripheral neuropathies.

Quinolones are bactericidal agents with a mechanism of action that involves direct inhibition of DNA synthesis. The first successful fluorination of a quinolone drug was in 1986, in the form of norfloxacin, which facilitated the crossing of the blood-brain barrier and making it more suitable for the treatment of central nervous system infections. Fluoroquinolones were widely prescribed given their pharmacokinetic and pharmacodynamics features.^1-3^

Hedenmalm and Spigset^4^ reported a series of 582 cases of acute drug reactions to fluoroquinolones that included 37 cases of acute toxicity of the peripheral nervous symptoms. Patients reported the following: paresthesia of the feet, legs, hands, and/or arms (n = 30, 81%); numbness/hypoesthesia (n = 10, 27%); pain/hyperesthesia; and muscle weakness (n = 4, 11%). In 6 cases (16%), the symptoms were unilateral. Concomitant symptoms included dizziness (n = 6), fatigue (n = 5), muscle cramps or convulsions (n = 3), local edema (n = 2), headache (n = 2), and gastrointestinal side effects (n = 2). Two patients had elevated liver enzymes. In one patient (No. 11), examination by electromyography and nerve conduction velocity because of persistent symptoms after 2 months revealed no sign of neuropathy.

Cohen^5^ also described peripheral nervous system symptoms among other types of toxicity that they attributed to fluoroquinolones and suggested a possible association between fluoroquinolone antibiotics and severe, long-term adverse outcomes in the peripheral nervous system and other organs. Ali et al,^6^ after analyzing cases reported to the FDA Adverse Event Reporting system between 1997 and 2012, described an association between fluoroquinolones and peripheral neuropathy and suggested a possible connection with more severe forms of nerve damage including Guillain-Barre syndrome.

Etminan et al^7^ quantified the risk of peripheral neuropathy in patients treated with oral fluoroquinolones. He found that current users, especially new users of fluoroquinolones, are at a higher risk of developing peripheral neuropathy and recommended that clinicians should weigh benefits against the risk of adverse events when prescribing these drugs to their patients, and highlighted that one of the challenges for a proper diagnosis of fluoroquinolone-associated peripheral neuropathy is the lack of hallmark clinical features in this patient population.

IVIG has been widely used for the treatment of peripheral neuropathy. Nowadays it is used as a first-line therapy in the treatment of Guillain-Barre syndrome, chronic inflammatory demyelinating polyneuropathy, and multifocal motor neuropathy.^8^

Diabetes mellitus is a well-known cause of peripheral neuropathy affecting 30% to 50% of patient with diabetes, depending on disease duration and glycemic control. Sharma and associates conducted an open trial where they studied patients with diabetic neuropathy and chronic inflammatory demyelinating polyneuropathy (CIDP) in their response to IVIG infusion with outcomes measured at 4 weeks from initiation of therapy, and the authors found a statistically significant improvement. In the case that we are presenting, we treated the patient with IVIG after other treatments failed.^2,9,10^

Currently, there are no guidelines for the treatment of peripheral neuropathy induced by fluoroquinolones, and the mechanism by which fluoroquinolones causes peripheral neuropathy remains elusive.^2^ This report illustrates the challenges presented by intractable pain in a patient that developed peripheral neuropathy after 10 days of treatment with a fluoroquinolone. This case is a word of caution in the use of fluoroquinolones in patients with diabetes mellitus type 1 as they might be at higher risk to develop peripheral neuropathy.^11^ This case also suggests a role for IVIG in the management of fluoroquinolone-induced peripheral neuropathy implying an underlying autoimmune mechanism.
